# Intracavernous Injection of Platelet-Rich Plasma Therapy Enhances Erectile Function and Decreases the Mortality Rate in Streptozotocin-Induced Diabetic Rats

**DOI:** 10.3390/ijms23063017

**Published:** 2022-03-10

**Authors:** Chun-Hou Liao, Kau-Han Lee, Shiu-Dong Chung, Kuo-Chiang Chen, Chellappan Praveen Rajneesh, Bo-He Chen, Jai-Hong Cheng, Wang-Ying Lin, Han-Sun Chiang, Yi-No Wu

**Affiliations:** 1 Department of Surgery, Cardinal Tien Hospital, New Taipei City 231, Taiwan; liaoch22@gmail.com; 2 School of Medicine, Fu Jen Catholic University, New Taipei City 242, Taiwan; kkhk88@gmail.com (K.-C.C.); praveenrajneesh@gmail.com (C.P.R.); s9122099s@hotmail.com (B.-H.C.); a17298674@yahoo.com.tw (W.-Y.L.); 3 Division of Urology, Department of Surgery, Chi-Mei Medical Center, Tainan City 71004, Taiwan; kauhan@hotmail.com; 4 Division of Urology, Department of Surgery, Far Eastern Memorial Hospital, New Taipei City 220, Taiwan; chungshiudong@gmail.com; 5 Department of Nursing, College of Healthcare & Management, Asia Eastern University of Science and Technology, New Taipei City 220, Taiwan; 6 Graduate Institute of Medicine, Yuan Ze University, Taoyuan City 320, Taiwan; 7 Department of Urology, Cathay General Hospital, Taipei City 106, Taiwan; 8 Center for Shockwave Medicine and Tissue Engineering, Kaohsiung Chang Gung Memorial Hospital and Chang Gung University College of Medicine, Kaohsiung 833, Taiwan; cjocko@gmail.com; 9 Medical Research, Kaohsiung Chang Gung Memorial Hospital and Chang Gung University College of Medicine, Kaohsiung 833, Taiwan; 10 Department of Leisure and Sports Management, Cheng Shiu University, Kaohsiung 833, Taiwan; 11 Graduate Institute of Biomedical and Pharmaceutical Science, Fu Jen Catholic University, New Taipei City 242, Taiwan; 053824@mail.fju.edu.tw; 12 Department of Urology, Fu Jen Catholic University Hospital, New Taipei City 242, Taiwan

**Keywords:** diabetes mellitus, erectile dysfunction, platelet-rich plasma, neuroregeneration

## Abstract

Erectile dysfunction (ED) is an agonizing complication of diabetes mellitus (DM) and it is challenging to treat ED in DM patients. Platelet-rich plasma (PRP) is a unique therapeutic strategy comprising intrinsic growth factors. An attempt was made to explore the potentiality of the PRP treatment in DM-induced ED rats in various groups (control, DM-non-ED, DM-ED, and DM-ED treated with PRP). Streptozotocin (STZ) was used to induce DM in rats. The blood glucose levels of the DM rats were maintained at >300 mg/dl. In the 18-week experiment, survival rate, body weight, intracavernous pressure (ICP) variations, and arterial blood pressure were analyzed. The tissue restoration results were validated by histological, immunofluorescence, and transmission electron microscopic analysis. PRP treatment of DM-ED rats significantly increased all parameters of erectile function compared to pre-treatment of PRP and DM-ED treated with vehicle. The histological results revealed that PRP treatment substantially enhanced the regeneration of myelinated nerves and decreased the atrophy of corporal smooth muscle. Notably, the PRP treatment immensely enhanced the survival rate in post-surgery DM-ED rats. These results indicated certain benefits of PRP treatment in delaying damage and preventing post-surgery complications in DM patients. Hence, PRP treatment is a novel multifactorial strategy for DM-ED patients.

## 1. Introduction

Erectile dysfunction (ED) is a predominant sexual dysfunction and is considered a key factor of functional incapability in sexual intercourse. In normal penile erection, the healthy nitrergic nerves, endothelium, and smooth muscles are the major components that play a vital role in the process [1]. Several factors such as drugs and surgery induce ED [2,3], and it has been emphasized as a common chronic complication of diabetes mellitus (DM) [4,5,6,7]. Recent studies revealed that 52.5% of DM patients have ED, thus implying that more than half of DM patients have sexual complications. Besides this, DM is analogous to ED pathophysiologically, involving endothelial dysfunction and pathological changes of vessels, muscles, and nerves [8,9]. The DM-induced ED is mainly triggered by the hyperglycemic condition, which leads to an overproduction of free radicals to increase oxidative stress and inflammation, thereby resulting in endothelial dysfunction of the corpus cavernosum [10,11]. Besides this, other complications such as vasculopathy, neuropathy, and endothelial dysfunction in DM patients lead to a more severe state of ED, making it more challenging to treat DM-ED than ED of non-diabetic patients [8,9].

As DM-ED can severely diminish quality of life [12], recent studies have focused on multiple dimensions of DM-ED treatment to elevate the quality of life. Numerous therapeutic measures for DM-ED are currently being practiced such as prosthesis, vacuum devices, surgical therapy, stem cell therapy, and the most frequently employed oral medical therapy [1,2]. The well-known drug phosphodiesterase 5 inhibitor is widely used for ED pharmacotherapy. Indeed, it has few side effects (for instance, headaches and gastrointestinal indisposition) and consequently limits patient compliance [13]. In recent years, stem cell-based treatments have been developed. These treatments tremendously decrease patients’ distress.

Nevertheless, stem cells possibly induce inflammatory reactions [1]. However, it is clinically and ethically challenging to implement stem cell-based treatments. Besides this, recent research has confirmed that exosomes derived from stem cells that have been treated for DM-ED effectively reduced inflammatory reactions and improved erectile function in rat models [14]. However, the preparatory process is complicated, cost-effective, and time-consuming. Hence, there are no effective management measures or permanent cure for DM-ED.

Recent studies and evidence from the available literature state that insufficient nerve growth factors and vascular endothelial growth factors in the blood are highly associated with DM-ED [2,9,15]. The platelet-rich plasma (PRP) is clinically proven with plenty of vital growth factors [14,16,17,18,19,20,21,22,23]. The indispensable part of the PRP is easy to collect and can separate the content from the blood with a simple centrifugation technique. Our previous studies have revealed that PRP has a neuroprotective effect and can facilitate the recovery of erectile function in the bilateral cavernous nerve injury rat model, based on reduced expressions of transforming growth factor-β1 (TGF-β1S) and caspase-3 [21,22]. Our subsequent study (in publication) indicated that PRP also has tissue- and smooth muscle-protective capabilities in cavernous nerve injury models. Hence, we have hypothesized that the properties of PRP could resolve the complex aetiology of DM-ED. Hitherto, no study has demonstrated the mechanism or potentiality of PRP treatment and treating it for DM-ED to reduce the ED complications. Hence, this study aimed to demonstrate PRP’s therapeutic potential and role in improving erectile function in a streptozotocin (STZ)-induced DM-ED rat model.

## 2. Results

### 2.1. Changes in Body Weight and Blood Glucose Level

**Figure 1:**
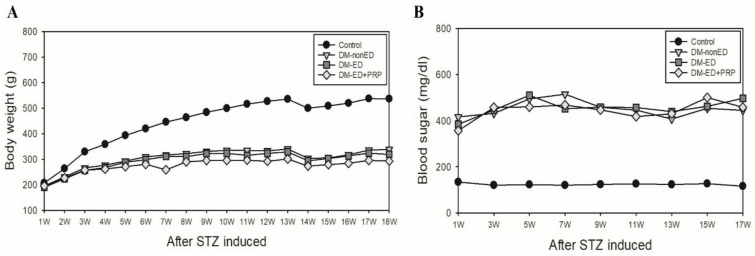
Graph showing the changes in body weight and blood glucose level of the normal control and experimental rats. (**A**) Graph showing the body weight changes of rats in each group and their respective control. (**B**) Graph showing the variations in the blood glucose levels of the rats in each group and their respective control. The body weight was recorded since the rats were six weeks old, and the streptozotocin (STZ) was also induced simultaneously, following blood sugar tested on the next week (7 weeks old). DM-non-ED, STZ-induced diabetes mellitus (DM) without erectile dysfunction (ED); DM-ED, STZ-induced DM with ED and treated with saline; DM-ED + PRP, STZ-induced DM with ED and treated with platelet-rich-plasma.

To determine the cumulative effect of STZ-induced hyperglycemic rats, we compared all of the experimental groups’ body weight and blood glucose levels and their respective normal controls. Alterations in the bodyweight are portrayed in Figure 1A. A decrease in the bodyweight of DM rats was observed compared with that of the control rats after one week of STZ treatment. In contrast, a trending weight gain in the control rats was also observed. After the first ICP measurement at 18 weeks, an overall slight decrease in body weight and a gentle gain back to stable body weight was observed in DM rats. The blood glucose level in DM rats was ˃300 mg/dL after one week of STZ treatment, and it was maintained until euthanasia. On the contrary, the non-diabetic rats showed stable blood glucose levels of <200 mg/dL (Figure 1B).

### 2.2. PRP Treatment Decreases the Mortality Rate

**Figure 2:**
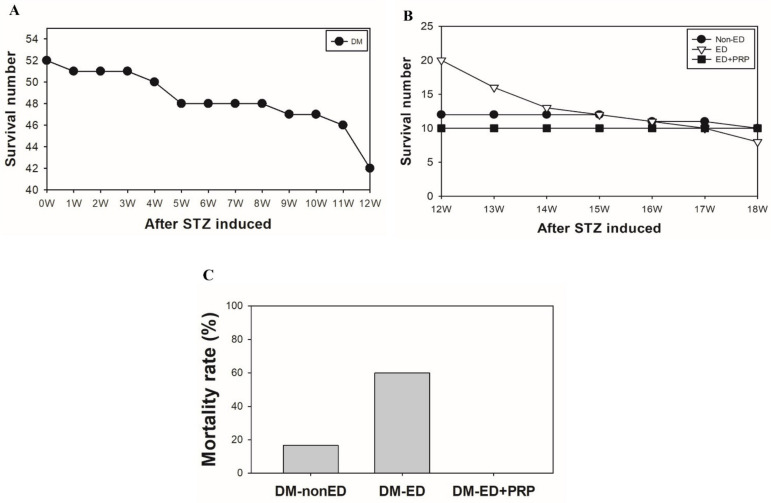
The graphical representation of the decreased mortality rate due to platelet-rich plasma (PRP) treatment. (**A**) Indicating mortality rate of rats after streptozotocin (STZ) induction until 12 weeks. (**B**) Showing the survival rate of the rats after the first ICP measurement and follow-up experiments. (**C**) Histogram showing the comparison among rats belonging to DM-non-ED, DM + ED, and DM + ED + PRP treatment. DM-ED, STZ-induced diabetes mellitus (DM) with erectile dysfunction (ED) and treated with saline (*n* = 20); DM-ED + PRP, STZ-induced DM with ED and treated with PRP (*n* = 10); vehicle-treated *n* = 12.

A total of 52 rats underwent the STZ induction, and 10 rats had died within 12 weeks of STZ induction (Figure 2A). The remaining 42 rats were divided into two groups: DM-ED (*n* = 30 rats) and DM without ED (*n* =12 rats) after the first ICP measurement. The DM-ED group was further divided into PRP treated group (*n* =10 rats) and the vehicle group (*n* = 20 rats). Remarkably, none of the rats had perished in the PRP treated group during the follow-up experiments, and the mortality rate was recorded as 0%. On the contrary, 12 rats had perished in the vehicle-treated group during the follow-up experiments, and the mortality rate was documented as high as 60% (12/20) (Figure 2B). Later on, the DM without ED and DM-ED treated groups were compared with the PRP group. In the follow-up observation period, the rats in the DM without ED group exhibited a 16.7% (2/12) mortality rate (Figure 2C). In summary, the PRP treatment efficiently reduced the effects of STZ toxicity and the adverse impacts of hyperglycemia in rats and simultaneously increased the survival rate of DM rats in the post-surgery state.

### 2.3. ICP Measurements after 12 Weeks of STZ Induction

**Figure 3:**
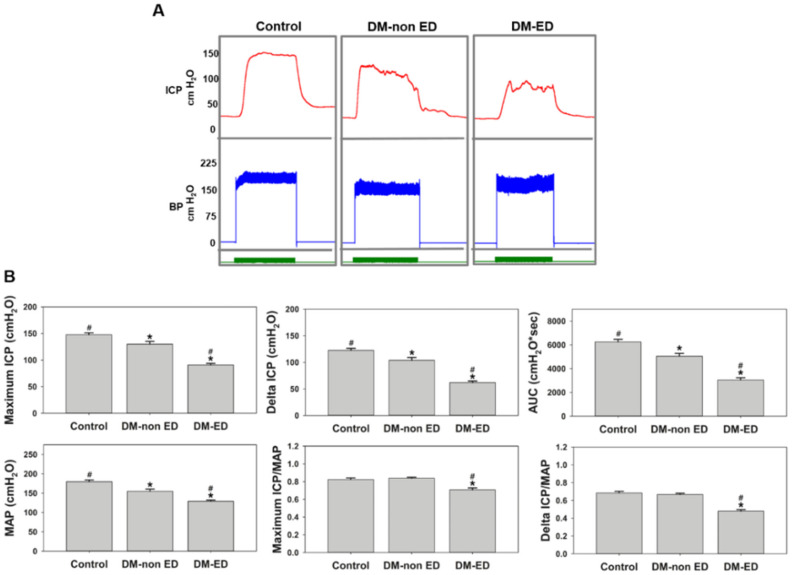
The erectile function parameters were compared after 12 weeks of streptozotocin (STZ) induction. (**A**) Recordings of intracavernous pressure (ICP) and blood pressure (BP) in control, DM non-ED, and DM-ED rats. (*n* ≥ 8) DM-non ED, STZ-induced diabetes mellitus (DM) without erectile dysfunction (ED); DM-ED, STZ-induced DM with ED and treated with saline. (**B**) The quantitative results of an erectile function parameter (Mean arterial pressure, ICP, and AUC) were calculated for each group (control, DM non-ED, and DM-ED rats). * *p* < 0.05 when compared with control, ^#^ *p* < 0.05 when compared with DM-non ED. (AUC—area under the curve; ICP—Intracavernous pressure; MAP—mean arterial pressure).

ICP measurements were performed after using STZ to induce a DM state for 12 weeks and simulate the DM-ED conditions. Figure 3 exhibits the first ICP results of rats from three groups (controls, DM-non-ED, DM-ED). The maximum ICP, delta ICP, AUC, and MAP values were the lowest in the DM-ED group compared to the three groups. Simultaneously, the MAP-normalised maximum ICP and delta ICP in the DM-non-ED group were similar to those of the control group. However, the values of the DM-ED group were remarkably lower than those in the other two groups. The erectile function of the DM-ED rats was about 60% of that of the normal. In other words, STZ injection decreased blood pressure, thereby inducing ED in the DM rats. The inferred data affirmed that the process of ED induction was successful in DM rats.

### 2.4. ICP Changes Due to Different Treatments

**Figure 4:**
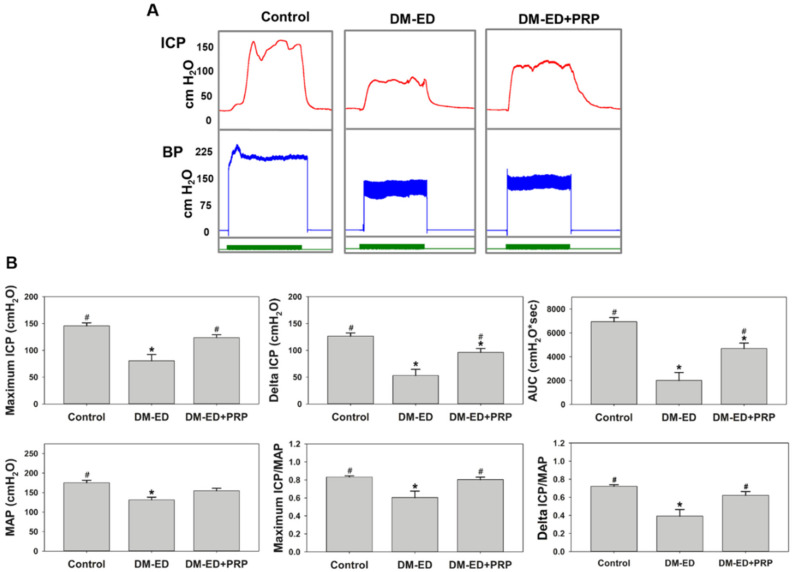
The erectile function parameters were compared after four weeks of platelet-rich plasma (PRP) or saline treatment. (**A**) Recordings of intracavernous pressure (ICP) and blood pressure (BP) in control, DM-ED, and DM-ED + PRP rats. Control, DM-ED, streptozotocin (STZ)-induced DM with ED and treated with saline; DM-ED + PRP, STZ-induced DM with ED and treated with PRP. (**B**) The quantitative results of an erectile function parameter (Mean arterial pressure, ICP, and AUC) were calculated for each group (control, DM-ED, and DM-ED + PRP rats). * *p* < 0.05 when compared with control, ^#^ *p* < 0.05 when compared with DM-ED. (AUC—area under the curve; ICP—Intracavernous pressure; MAP—mean arterial pressure).

The results of erectile function assessment after four weeks of PRP or saline treatment are displayed in Figure 4. PRP treatment seemingly regained the BP, albeit no statistical difference was observed. Nevertheless, the BP of the DM-ED rats remained significantly lower than that of the control rats. The maximum ICP, delta ICP, and AUC were substantially lower in the DM-ED group than in the other three groups. On the contrary, the maximum ICP, delta ICP, and AUC could be improved by PRP treatment. The MAP-normalised maximum ICP and delta ICP were significantly lower in the DM-ED group (maximum ICP/MAP = 0.60 and delta ICP/MAP = 0.39) than in the other groups. In contrast, these values were remarkably higher in the DM-ED + PRP group than in the DM-ED group, similar to the DM-non-ED group, with the maximum ICP/MAP = 0.80 and delta ICP/MAP = 0.62.

**Figure 5:**
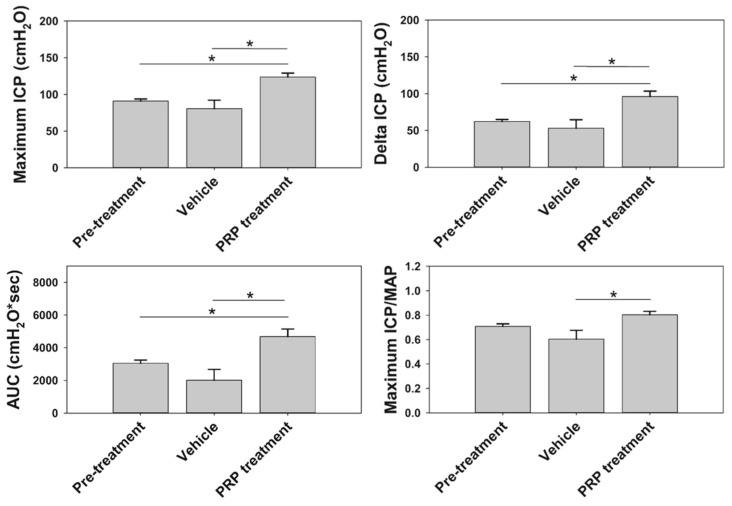
The illustration shows the comparison of erectile function parameters of streptozotocin (STZ)-induced diabetes mellitus (DM) rats. Pre-treatment, after 12 weeks of STZ-induced DM, 18 weeks old (*n* = 8); vehicle, STZ-induced DM with erectile dysfunction (ED) and treated with saline, and ICP test performed after saline treatment for four weeks (24 weeks old) (*n* = 7); platelet-rich-plasma (PRP) treatment, STZ-induced DM with ED and treated with PRP, and ICP test performed after PRP treatment for four weeks (24 weeks old) (*n* = 8). * *p* < 0.05 when compared with PRP treatment. (AUC—area under the curve; ICP—Intracavernous pressure; MAP—mean arterial pressure).

Simultaneously, the ICP and BP of the pre-treated DM-ED group and vehicle group were compared with the DM-ED + PRP treatment group for effect assessment (Figure 5). The values in the vehicle group indicated a slight decrease (though not significantly different), suggesting that the decline in erectile function was dependent on the duration of DM. In contrast, the treatment group had distinctively higher values than the vehicle group. These data insinuated that PRP treatment could improve erectile function in DM rats.

### 2.5. PRP Treatment Restored the Damaged Corpus Cavernosum

**Figure 6:**
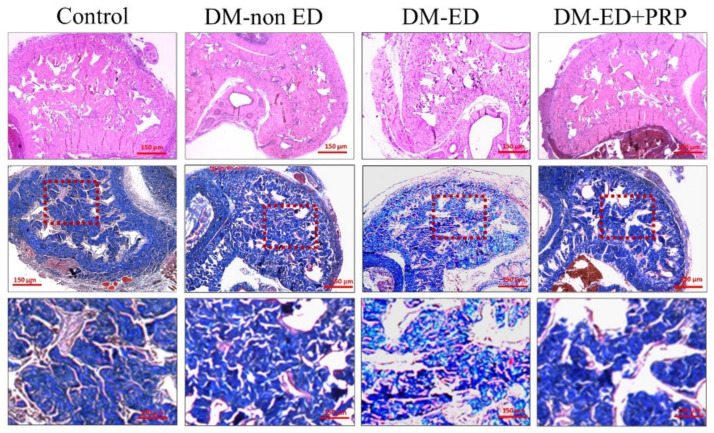
Micrograph showing the hematoxylin and eosin staining and Masson’s trichrome staining of the corpus cavernosum (40× magnification). DM-non ED, streptozotocin (STZ)-induced diabetes mellitus (DM) without erectile dysfunction (ED); DM-ED, STZ-induced DM with ED and treated with saline; DM-ED + PRP, STZ-induced DM with ED and treated with platelet-rich-plasma. Red colour demarcated area magnified into 200×.

The results of the H&E and Masson’s trichrome staining in the corpus cavernosum are illustrated in Figure 6. The H&E staining indicated varying degrees of damage in the DM-non-ED and DM-ED groups, along with inflammations in the DM-ED group. Similar observations were made from Masson’s trichrome staining, where the structure of the smooth muscles was also severely damaged in the DM-ED group. After PRP treatment, the histological patterns of the corpus cavernosum were restored to be similar to those observed in the DM-non-ED group.

**Figure 7:**
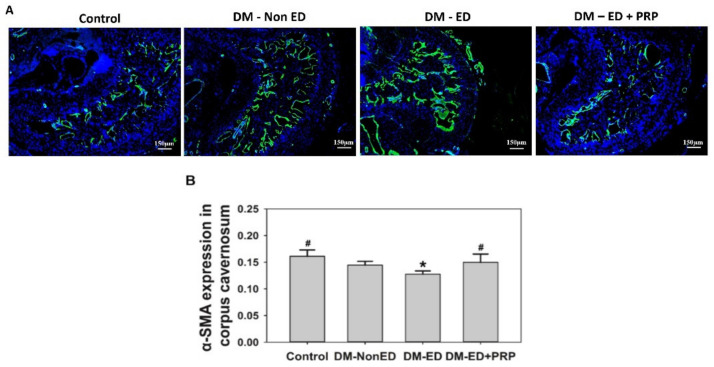
(**A**) Image showing the Immunofluorescence results of α-smooth muscle actin (α-SMA); (**B**) expression of α-SMA in the corpus cavernosum. DM-non ED, streptozotocin (STZ)-induced diabetes mellitus (DM) without erectile dysfunction (ED); DM-ED, STZ-induced DM with ED and treated with saline; DM-ED + PRP, STZ-induced DM with ED and treated with platelet-rich-plasma. * *p* < 0.05 when compared with control, ^#^ *p* < 0.05 when compared with DM-ED.

The immunofluorescence results (Figure 7A) confirmed an improved condition in the corpus cavernosum after PRP treatment, and the statistical analysis also portrayed a similar condition (Figure 7B).

**Figure 8:**
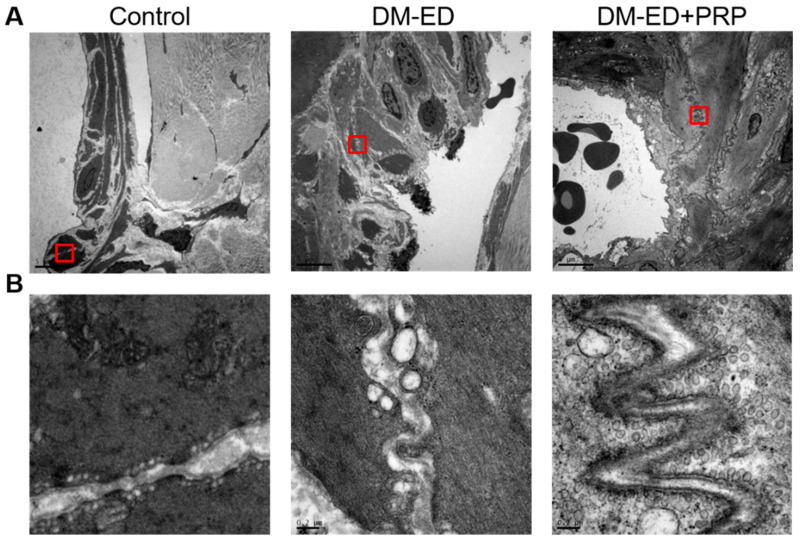
Transmission electron microscopy (TEM) of the caveolae in 2500× (**A**) and 40,000× (**B**) magnifications of the red-coloured demarcated area from (**A**). DM-ED = streptozotocin (STZ)-induced diabetes mellitus (DM) with erectile dysfunction (ED) and treated with saline; DM-ED + PRP, STZ-induced DM with ED and treated with platelet-rich-plasma. (Scale: (**A**): 5 µm; (**B**): 0.2 µm).

These results indicated that DM could reduce α-SMA expression radically since the lowest expression of α-SMA was observed in the DM-ED group. Nevertheless, a significant and increased expression of α-SMA was documented after PRP treatment. Moreover, the TEM analysis of the caveolae of smooth muscle in the corpus cavernosum revealed that the distribution of caveolae was considerably decreased in the DM-ED rats compared to normal control, and the presence of caveolae was abundantly detected after PRP treatment (Figure 8). The high incidence of caveolae affirmatively indicated an improved function of smooth muscles, and these data profoundly suggested that damage to the corpus cavernosum due to DM was undeniably improved by the PRP treatment.

### 2.6. PRP Treatment Reversed Nerve Damage

**Figure 9:**
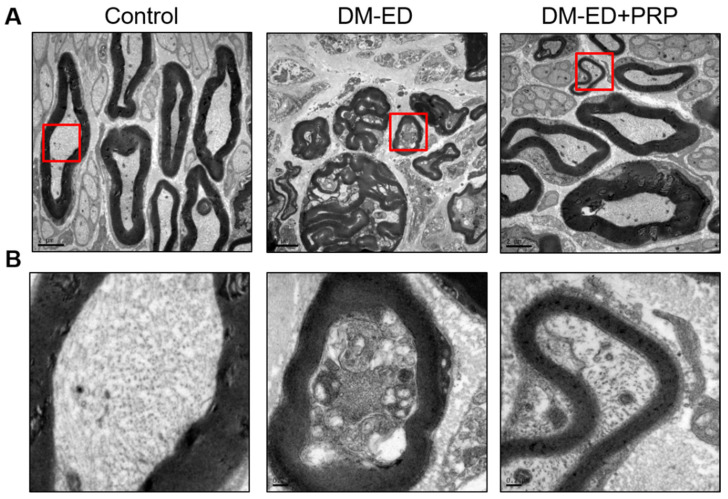
Transmission electron microscopy (TEM) of the cavernous nerve in 5000× (**A**) and 30,000× (**B**) magnifications of the red-coloured demarcated area from (**A**). DM-ED, streptozotocin (STZ)-induced diabetes mellitus (DM) with erectile dysfunction (ED) and treated with saline; DM-ED + PRP, STZ-induced DM with ED and treated with platelet-rich-plasma. (Scale: (**A**): 2 µm; (**B**): 0.2 µm).

A healthy cavernous nerve might be considered essential for normal erectile function. The TEM results of the cavernous nerve are depicted in Figure 9. The DM-ED rats exhibited a thinner myelin sheath with an irregular arrangement plane. Additionally, the observed myelinated axons were comparatively smaller and fewer in numbers than the normal control. Furthermore, the axon cells of the DM-ED rats displayed several abnormal endosomes and fewer irregular mitochondria. The PRP treatment substantially improved these anomalies, including axon size, myelin cell thickness, arrangement regularity, mitochondrial normality, and myelinated nerve regeneration. The PRP treatment displayed an upturn in nerve damage and potentially induced neuroregeneration.

## 3. Discussion

ED is a general and notable complication in DM [4,5,6] and a challenging task to treat as a consequence of its heterogeneous nature [1,4,5,6,8,9]. This study provided positive evidence that PRP was a multifunctional treatment for ED in STZ-induced DM rats, including a decrease in mortality of STZ toxicity and hyperglycemia, increase in wound healing of post-surgery, recovery of ICP, smooth muscles atrophy prevention, and nerves regeneration. The model of STZ-induced DM rats was widely employed to study the pathology of diabetes and the ailments caused by it. The following challenge was encountered in determining the STZ dosage while establishing the DM model prior to the treatment process. While low doses (<60 mg/kg) of STZ induced reversible hyperglycemia, higher doses; >75 mg/kg STZ in rats or >200 mg/kg STZ in mice; induced mortality of the animal [24,25,26]. In this study, 65 mg/kg STZ was used, and no mortality was documented after STZ induction within a week. However, the survival rate of STZ-induced DM rats was 43% throughout the study. The mortality was consistent with previous studies, which indicated a 20–50% mortality in 65 mg/kg STZ-induced albino rats [27,28,29]. The causes of STZ induced mortality are typically attributed to its toxicity and pathophysiological progress of hyperglycemia. In general, STZ triggered cardiac and adipose tissue damage, elevated oxidative stress, inflammation, and endothelial dysfunction [30]. Besides this, an increased death toll was documented in STZ treated rats due to pulmonary edema and renal abscess [31]. A previous study also stated that slower wound healing in DM could lead to gangrene and death [32]. These results reflect DM patients’ potential clinical complications in their day-to-day lives. A previous study highlighted a low survival rate in STZ-induced DM mice, and a 20% improvement in survival rate could be observed after softened food intake [33]. A recent report stated that IP or oral administration of 230 mg/kg nicotinamide before STZ treatment increased the survival rate [34]. The present study documented the survival rate at 100% in the DM-ED with PRP treatment group since the PRP has higher concentrations of thrombocytes and growth factors that augment the tissue, nerve repair, and regeneration [35]. In this study, we have simulated several clinical conditions similar to diabetic patients, comprising hyperglycemia with and without ED, hyperglycemia with ED and abdominal surgery induction, and hyperglycemia with ED and treated with PRP during surgery. Herein, the mortality scenario of hyperglycemia with ED rats treated with PRP during surgery had exhibited a significantly lower level of mortality rate than other groups. These findings indicate certain benefits of PRP treatment in decreasing the inflammatory injuries caused by surgery in DM patients. Albeit, the molecular mechanism behind this event is still elusive. Perhaps the wound-healing nature and improvement in tissue regeneration due to PRP treatment might improve DM rats’ survival rate.

It is prominent that the erectile function in DM-ED rats has been improved after PRP treatment. As shown in Figure 3 and Figure 4, the erectile function was higher in the non-treated and pre-treatment groups by 1.58- and 1.29-fold, respectively, and the maximum ICP was 123.68 cmH_2_O (90.97 mmHg). Recently, numerous studies have focused on the cell-based treatment for DM-ED. A contemporary study determined that the maximum ICP of bone marrow mesenchymal stem cell treatment was ~50 mmHg. When combined with stromal cell-derived factor-1 therapy, the maximum ICP was ~75 mmHg [36]. However, as we mentioned earlier, stem cell-based treatment could potentially induce inflammation, such as an enhanced NF-κB expression in DM-ED rats [1]. Another related study demonstrated that using exosomes of adipose-derived stem cells for DM-ED therapy achieved a maximum ICP of ~55 mmHg [14]. PRP has the best treatment effect for DM-ED rats compared with the aforementioned stem cell-based treatments. Besides, cell-based treatments possess ethical concerns and are complicated in preparatory and transfer techniques [37,38,39,40]. However, notably, no such legal statements were needed for autologous PRP treatment.

Previous findings suggested that CN played a significant role in nerve-regulating erectile function, and surgical mishaps in CN induce corporal fibrosis and ED [21,22,41,42]. CN damage and smooth muscle loss were also observed in this study, especially in the DM-ED group. Nerve damage is a complicated event and is hard to rectify in DM. Based on our previous studies, PRP can downregulate the expressions of TGF-β1 and caspase-3 and induce an outstanding level of platelet-derived growth factor for neuroprotection and neuroregeneration in surgical CN injury [21,22]. Neuroprotection and neuroregeneration were also observed in the DM-ED + PRP group, denoted by the larger myelinated axon size, even myelin cell thickness, increased number and regular arrangement of myelin, and generation of new healthy nerves. Smooth muscle improvement in the corpus cavernosum by PRP treatment could be deduced based on the presence of abundant caveolae. Caveolae are abundant in endothelial and smooth muscle cells and are also found in the smooth muscles of the corpus cavernosum in mice and rats. As caveolae have been considered to improve erectile function, many caveolae were conjectured to be associated with better erectile function. For instance, aged rats were found to have fewer caveolae in the smooth muscle than younger rats [41,42].

## 4. Materials and Methods

### 4.1. Experimental Animal 

In total, 62 male Sprague-Dawley (SD) rats (5-week-old when purchased) (BioLasco Taiwan Co., Ltd., Taipei, Taiwan) were used in this study. The study was approved by the Fu Jen Catholic University Institutional Animal Care and Use Committee (approval no.: A10568 dated: 3 May 2017). All of the study protocols and procedures followed in the present study were performed under the recommended guidelines.

### 4.2. Experimental Design

**Figure 10:**
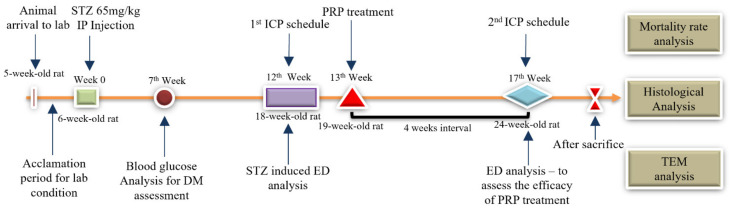
Timeline indicating the experimental schedule of the normal control and DM-ED rats. (TEM-transmission electron microscope; ED-erectile dysfunction; ICP-intracavernous pressure; STZ-streptozotocin; PRP-plasma rich plasma).

The rats were separated at random into two groups: control (10 rats) and STZ-induced groups (52 rats). The STZ-induced group was treated with 65 mg/kg STZ (Sigma-Aldrich, Milwaukee, WI, USA) by intraperitoneal (IP) injection at 6 weeks old. After 12 weeks (18 weeks old), the intracavernous pressure (ICP) and arterial blood pressure (BP) of all of the rats were measured. According to the measured maximum ICP and mean arterial blood pressure (MAP), the STZ-induced rats were further separated into DM-non-ED (ICP ≥ 90 cmH_2_O or MAP ≥ 140 cmH_2_O) and DM-ED (ICP < 90 cmH_2_O or MAP < 140 cmH_2_O) rats. The DM-ED rats were randomly assigned to PRP or saline treatment and were treated with 200 μL PRP (DM-ED + PRP group) or saline solutions (DM-ED group) by intracavernous injection. After treatment for four weeks (17 weeks old), the ICP and BP of all of the rats from the four groups (control, DM-non-ED, DM-ED, and DM-ED + PRP) were analyzed. Concurrently, body weight was recorded weekly after domesticating for one week, and blood glucose (blood glucose of DM rats > 300 mg/dl) was measured fortnightly after one week of STZ treatment (Figure 10).

### 4.3. PRP Preparation

PRP preparation was performed according to our previous study [22]. In brief, the blood sample was collected from 10 healthy SD rats in a blood collection tube with anticoagulant citrate dextrose solution by coeliac arterial blood collection method. The blood samples were further centrifuged at 400× *g* for 30 min, and the supernatant was transferred into a new tube and subsequently centrifuged at 1500× *g* for 15 min. The PRP solution was pipetted out and stored at −20 °C for 15 days. The PRP concentration was detected using a TC10 automated cell counter (Bio-Rad, Berkeley, CA, USA), and the concentration was later adjusted to 1.5 × 10^6^ platelets/μL.

### 4.4. Assessment of Erectile Function

The ICP and BP of rats were measured to evaluate erectile function. After exposing the penis, the cavernous nerve (CN) was identified. For finding the ICP value, a polyethylene-50 syringe (24G needle) was filled with 50 U/mL heparin solution. A needle was inserted into the right crus of the penis near the CN, which was connected with an MP36 pressure transducer (Biopac Systems Inc., Goleta, CA, USA) and recorded with BSL 4.0.3 software. A stainless-steel bipolar electrode was used to stimulate the CNs directly. The parameters of monophasic rectangular pulses were set at 5-mA amplitude, 20-Hz frequency, 0.2-ms pulse width, and 60 s duration, performed by a DS3 constant current isolated stimulator (AutoMate Scientific Inc., Berkeley, CA, USA). Maximum, minimum, and delta ICP areas under the ICP curve and MAP were analyzed.

### 4.5. Surgical Procedures

The rats were anesthetized (IP) by sodium pentobarbital (40 mg/kg). After shaving the surgical site, an iodine-based solution was applied for sterilization, and an incision was made at the lower abdomen. The penises of the DM-ED rats were exposed and treated with 200 μL saline or PRP solutions accordingly, by corpus cavernosum injection using a 0.5 mL insulin syringe with a 30G needle (Becton Dickinson, San Diego, CA, USA). The DM-non-ED group did not receive any treatment after the abdominal opening. The abdominal incision was finally closed with a single-layer suture.

### 4.6. Histology and Immunofluorescence Staining

After administering an overdose of pentobarbital sodium solution, the rats were sacrificed by coeliac arterial blood collection. The middle portion of the penis was collected and immediately fixed with 10% formaldehyde (*w*/*v*). After 24 h, the tissues were embedded in paraffin blocks, and the paraffin-embedded tissues were sliced at 5 μm thickness for hematoxylin and Eosin (H&E), Masson’s trichrome, and immunofluorescence staining. Before staining, the segments were dewaxed in xylene for 10 min, and the process was repeated twice. The tissues were hydrated with a graded concentration of ethanol (100%, 90%, 80%, and 70%) and double-distilled H_2_O for 5 min. For the immunofluorescence staining process, the slides were first soaked in blocking solution (10% goat serum, 2% bovine serum albumin, and 0.2% Triton X-100) (Sigma-Aldrich, St. Louis, MO, USA) for 1 h at room temperature. The primary antibodies for α-smooth muscle actin (α-SMA) (Abcam Inc., Cambridge, MA, USA), neuronal nitric oxide synthases (nNOS) (Santa Cruz Biotechnology, Santa Cruz, CA, USA), and β III tubulin (Abcam Inc., Cambridge, MA, USA) were incubated with the segments at 4 °C overnight. After removing the primary antibodies, a secondary antibody conjugated to Alexa Fluor 488 (Invitrogen, Carlsbad, CA, USA) (1:400 dilution) was incubated with the samples for 1 h at room temperature. DAPI staining was performed for nuclear colocalization. Finally, the immunohistochemistry results were obtained by fluorescence microscopy, and computerized histomorphometric analyses were performed using ImageJ software (National Institutes of Health, Bethesda, MD, USA).

### 4.7. Transmission Electron Microscopy (TEM)

Initially, the tissues were cut into 2 mm segments and fixed with 2.5% phosphate-buffered glutaraldehyde (0.1 M, pH 7.2) for the TEM analysis. A 1% phosphate-based osmium tetroxide buffer (0.1 M, pH 7.2) was used for post-fixing. The segments were dehydrated with graded concentrations of alcohol and embedded in Epon-812. Semi-thin sections of 1 μm were sliced and stained with toluidine blue, while ultra-thin sections of <1 μm were sliced and stained with lead citrate and uranyl acetate. A JEOL JEM-1400 transmission electron microscope (JEOL, Tokyo, Japan) was used for image analysis.

### 4.8. Statistical Analysis 

All statistical analyses were performed using SPSS v.18.0 (SPSS Inc., Chicago, IL, USA), and the data was determined as means ± standard deviations. The divergence of ICP was performed using Pearson’s correlation analysis, and the one-way analysis of variance (ANOVA) was used to compare the differences between each group. Statistical significance was defined as *p* < 0.05 for all comparisons.

## 5. Novelty and Application Potentials of PRP in DM-ED

The execution of PRP treatment for various sports injuries and surgical procedures is relatively increasing even though it is rare and novel in the case of the DM-ED model, and it is a pioneer attempt. Evidently, DM increases the complexity of ED. The outcome of the present study indicates that PRP treatment effectively increases erectile function in DM-ED rats predominantly by enhancing the nerve regeneration and corporal smooth muscle proliferation processes. Besides this, the PRP treatment efficiently participates in the wound healing process and aids in faster recovery in post-surgery episodes of the DM-ED rats. In diabetic conditions, the wound healing process might be delayed for several reasons, principally due to suppressed immune response and poor blood circulation. Since the PRP is enriched with growth factors, it boosts wound healing and prevents ulcers. PRP treatment reduces the mortality of the rats after surgery induction by reducing the inflammation condition in the body cells. Initially, the work was designed to improve the erectile function with PRP treatment. Surprisingly, we have identified that apart from improving the erectile function, the PRP administration became a life-saving strategy after the surgical episode in the DM-ED rats. It is evident from the current findings that PRP treatment certainly might be beneficial not only for DM-ED patients but also for DM and other patients who might have been expecting aciurgy for their ailments. Remarkably it is highly recommended due to its reduced cost-effective condition and simple preparatory procedure. Since it is autologous, it rules out ethical issues and is pathogen-free. Apparently, PRP is a great boon for mankind if used appropriately.

## 6. Conclusions

In conclusion, this is a pioneer study to report the application of PRP in DM-ED treatment and has highlighted the remarkable potential of PRP in DM-ED treatment. We had provided evidence that the erectile function could be improved with PRP treatment by accelerating nerve regeneration and preventing corporal smooth muscle atrophy. Besides this, the PRP treatment tremendously enhanced the survival rate in DM-ED rats after surgery induction. These outcomes indicate certain benefits of PRP treatment in delaying the harm of hyperglycemia in DM patients. Further in-depth studies are warranted in this new dimension. PRP treatment certainly is a state-of-the-art strategy to prevent complications in diabetes patients who undergo clinical surgery in the future.

## Data Availability

The datasets of the present study can be available from the corresponding author upon request.
